# Protein structure alignment by Reseek improves sensitivity to remote homologs

**DOI:** 10.1093/bioinformatics/btae687

**Published:** 2024-11-15

**Authors:** Robert C Edgar

**Affiliations:** Independent Scientist, Corte Madera, CA 94925, United States

## Abstract

**Motivation:**

Recent breakthroughs in protein fold prediction from amino acid sequences have unleashed a deluge of new structures, presenting new opportunities and challenges to bioinformatics.

**Results:**

Reseek is a novel protein structure alignment algorithm based on sequence alignment where each residue in the protein backbone is represented by a letter in a “mega-alphabet” of 85 899 345 920 (∼10^11^) distinct states. Reseek achieves substantially improved sensitivity to remote homologs compared to state-of-the-art methods including DALI, TMalign, and Foldseek, with comparable speed to Foldseek, the fastest previous method. Scaling to large databases of AI-predicted folds is analyzed. Foldseek *E*-values are shown to be under-estimated by several orders of magnitude, while Reseek *E*-values are in good agreement with measured error rates.

**Availability and implementation:**

https://github.com/rcedgar/reseek.

## 1 Introduction

Understanding proteins is a foundational project of molecular biology, where forensic traces of evolution provide vital clues for inferring function and mechanism. Vast collections of protein amino acid (a.a.) sequences have been generated by low-cost next-generation generation sequencing, leading to millions of high-quality predicted structures enabled by recent breakthroughs in machine learning. Exploiting this new trove of data challenges computational methods developed in a previous era where solved structures numbered in the tens of thousands.

Popular pair-wise protein structure alignment algorithms include DALI (Holm and Sander 1993), TMalign (Zhang and Skolnick 2005), and Foldseek (Van Kempen *et al.* 2024). DALI and TMalign have highest accuracy according to recent benchmark tests (Holm 2019, Van Kempen *et al.* 2024), while Foldseek has high reported accuracy at speeds thousands of times faster.

In practice, a biologist must choose a score cutoff to make a trade-off between sensitivity and errors. The gold standard solution, pioneered by BLAST (Altschul *et al.* 1990), is to report an *E*-value, i.e. an estimate of the expected number of false positives that would be obtained by setting the cutoff at the same score as the alignment. Accurate *E*-values are vital for reliable search of large databases; their accuracy can be benchmarked by comparison with measured false-positive errors per query (*FPEPQ*).

**Figure 1.**
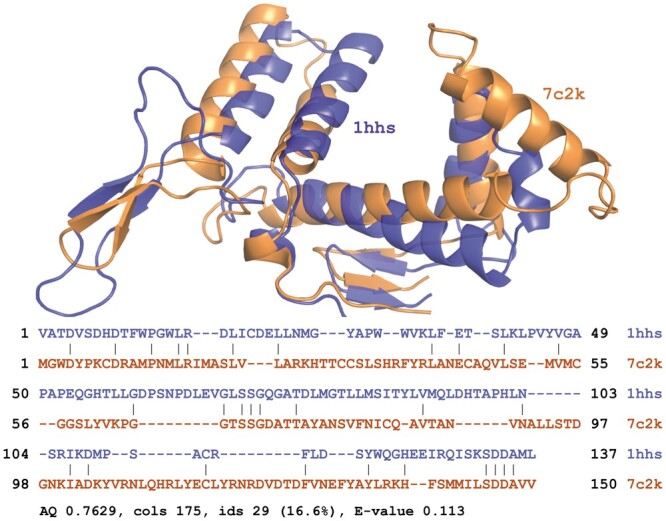
Reseek alignment of highly diverged virus polymerases. Rigid structure superposition (top) and amino acid sequence alignment (bottom) for structures 1hhs bacteriophage phi6 polymerase and 7c2k COVID-19 polymerase.

The conceptually simplest approach to aligning two protein structures is to seek a rigid-body transformation of one backbone which minimizes the root mean square distance (RMSD) of corresponding Cα atoms (Kabsch 1976, Umeyama 1991), see Fig. 1. This works well for close relatives. However, for more distantly related proteins, three problems arise: (i) similarity may be limited to a sub-segment of one or both structures so that local rather than global alignment is required, (ii) some regions may “flex” relative to others over evolutionary time so that somewhat different transformations should be applied to different segments, and (iii) the number of Cα atoms varies due to insertion and deletion mutations, which cases equivalences between residues, and hence alignments, to be ambiguous. Many different objective functions have been proposed for optimizing local alignments of Cα coordinates, including the DALI Z-score, TM-score, and CE (Shindyalov and Bourne 1998).

An alternative approach is to represent a structure as a sequence of letters, one for each a.a, and adapt sequence alignment algorithms such as Smith–Waterman (Smith and Waterman 1981) or BLAST. This approach was pioneered by 3D-BLAST (Yang and Tung 2006) and CLePAPS (Wang and Zheng 2008), which used conformational alphabets with 23 and 17 letters, respectively. In this type of alphabet, a letter represents the local conformation (secondary structure) of a Cα; e.g. if the Cα is in an alpha helix its letter might be H. Local conformation can be described by real-valued features including angles and distances which are invariant under rigid translation and rotation, i.e. independent of the arbitrary choice of axes for Cα coordinates (Hovmoller *et al.* 2002). Features for a reference set of Cαs are clustered to obtain a representative subset, and a letter for a given Cα is assigned by identifying the closest representative. In this way, continuous parameters characterizing local conformation are condensed into a discrete alphabet *A* with |*A*| ∼ 20 letters. The score of an alignment is the sum of log-odds scores for aligned pairs of letters, minus gap penalties, where the symmetrical |*A*|×|*A*| matrix *M^A^* of scores for every possible pair of letters is pre-trained on a reference set of trusted alignments. Training of this matrix uses methods developed for a.a. substitution matrices such as BLOSUM62 (Henikoff and Henikoff 1992).

Foldseek extends this approach from secondary to tertiary structure by considering the conformation of a Cα and its nearest neighbor by Euclidean distance. Their combined conformation is represented by a 10D feature vector and condensed into a 20-letter alphabet called 3Di. The score of an aligned pair of Cαs is calculated as a weighted sum of the log-odds score for their 3Di letters and the BLOSUM62 log-odds score of their corresponding a.a.s. The relative weight of scores for 3Di and a.a. letters is a constant parameter of the algorithm fixed by training on a reference set.

Intuitively, it seems clear that a great deal of information must be lost when backbone coordinates are condensed into a sequence of letters from an alphabet of size ∼20 letters. Consider Foldseek’s 3Di, which represents the conformation of a Cα and its nearest neighbor. Secondary structure can be condensed to an alphabet with four states {*helix, strand, turn, loop*}, which already loses information about beta strands (e.g. are they parallel or anti-parallel?), and lumps a rich variety of loop conformations into a single state. With one residue plus its neighbor, this gives 4 × 4 = 16 states, leaving little room to capture much more information in a 20-state alphabet. The Foldseek paper investigated the relationship between alphabet size and sensitivity in 3Di, where Supplementary Fig. S13 shows that a point of diminishing returns was reached at ∼20 letters. I believe this reflects increased difficulty in estimating the scoring matrix from a relatively small training set rather than an inherent limit to the power of larger alphabets. The log-odds score *M^A^_i,j_* for aligning letter *i* to letter *j* in alphabet *A* is calculated as

(1) $$M_{i,j}^{A}=\log\left(P_{i,j}/{(p}_{i}p_{j})\right),$$

where *P_i,j_* is the frequency of (*i, j*) as a fraction of all aligned pairs in the training set, and *p_i_* is the frequency of letter *i*. The frequency distribution *p_i_* is likely to be skewed with a tail of rare letters. With larger alphabets, the numbers of observed aligned pairs of rare combinations become too small to obtain an accurate estimate of joint probabilities *P_i,j_*, *i *≠* j* for low-frequency letters. Reseek solves this problem, thereby enabling arbitrarily large structure alphabets to be deployed in practical applications, by factoring structure states into tractable components whose log-odds scores are calculated independently.

## 2 Materials and methods

Reseek represents a Cα backbone atom and its structural context as a feature vector (FV) which may have many components. A component may be a single real value (scalar), multiple real values (vector), or inherently discrete. One component is the amino acid type (*AA*), an inherently discrete feature which can be represented by the standard 20-letter alphabet. Examples of real-valued features include *DistNEN*, the distance in Angstroms between the Cα at position *i* and its nearest Euclidean neighbor (NEN, i.e. the neighbor as defined by Foldseek), and *DistREN*, defined as the distance to the nearest neighbor in the opposite direction in the chain compared to its NEN (its reverse Euclidean neighbor, REN). The local conformation (*Conf*) at position *i* in the backbone is represented by real-valued features comprising all-vs-all pair-wise distances between the 2*κ *+ 1 positions centered at *i*, excluding adjacent pairs which are at an effectively constant distance set by the Cα−Cα bond length. By default, *κ *= 3. Similarly, the local context around the NEN and REN for position *i* is represented by all-vs-all pair-wise distances between the 2*κ *+ 1 positions centered at *NEN_i_* and *REN_i_*, respectively. For alignment construction and scoring, the FV is condensed into a discrete feature vector (DFV) in which each component is a letter from an alphabet which is typically chosen to have size ≲20 to mitigate problems with sparse training data for low-frequency letters. The procedure for condensing a feature depends on whether it is a scalar or vector.

Scalar features are condensed by collecting feature values for all Cαs in the training set and sorting by increasing numerical value. If the desired alphabet size is *L* letters, the sorted list is divided into *L* contiguous bins of equal size, and *L *−* *1 threshold values are calculated which divide the bins. To achieve this, the threshold *t_B_* between bin *B* and bin *B *+* *1 is set to the mean of the maximum value in bin *B* and the minimum value in bin *B *+* *1. The condensed alphabet letter of feature value *x* is then *argmin*(*B*: *x* > *t_B_*), i.e. the lowest bin for which *x* is above the threshold. Thus, letter assignment is reduced to comparison with *L *−* *1 thresholds. This procedure is designed to ensure that letters have approximately equal frequencies, maximizing the entropy of the alphabet and thereby maximizing the information in a sequence (Strait and Dewey 1996).

Vector features are condensed by collecting values for all Cαs in a training set of structures and clustering by *K*-means (MacQueen 1967), where *K* was set to the desired alphabet size *L*. Following training and storage of the cluster representatives, the letter for a feature vector is assigned by finding the closest representative. For search, this procedure for assigning letters may be more efficient than the corresponding procedure for the 3Di alphabet, which requires a neural network classifier at runtime.

Log-odds score matrices for discrete features, including the amino acid type, are calculated per Equation (1), where probabilities are estimated as the frequencies observed in a training set of alignments. The training set was constructed using TMalign from a subset of SCOP40 pair-wise alignments with 0.6 < *TM* < 0.8.

Let the discrete feature vector for the Cα at position *i* in a given protein be *V*(*i*), with integer-valued components (letters) *V_f_* (*i*), *f* ∈ **F** where **F** is the chosen set of features. The score for an aligned pair of positions *i, j* is calculated as

(2) $$S_{i,j}=\sum_{f}w_{f}M_{f}\left[V_{f}\left(i\right),V_{f}\left(j\right)\right],$$

where *M_f_* is the log-odds score matrix for feature *f*, and *w_f_* is its relative weight. A DFV has $h=\prod_{f}\left|A_{f}\right|$ possible states, where *A_f_* is the alphabet for feature *f* and a given DFV may therefore be regarded as a letter in a “mega-alphabet” *H* with *h* “mega-letters.” From this perspective. Equation (2) is an estimate of the log-odds score for a pair of mega-letters that would be obtained with a sufficiently large training set. The score of an alignment of proteins *Q* and *T* is

(3) $$s\left(Q,T|C\right)=\sum_{c}S\left[Q\left(k\right),T\left(k\right)\right]-\sum_{g}\{G_{\mathrm{open}}+\left(len_{g}-1\right)G_{\mathrm{ext}}\},\;$$

where **C** is the set of columns in the alignment, *c ∈* **C** is a column, *Q*(*c*) is the position in *Q* that appears in that column, *T*(*c*) is the position in *T*, *S* is calculated according to Equation (2), *g* is a gap in the alignment, *len_g_* is the number of columns in the gap, *G_open_* is the gap-open penalty, and *G_ext_* is the gap-extension penalty. A high-scoring local alignment is found using Smith–Waterman or a BLAST-like method which seeks to maximize *s* as given by Equation (3). *AA* is combined with eight 16-letter structural features, and the size of the mega-alphabet is therefore 20 × 16^8^ = 85 899 345 920 states by default.

A test statistic is a numerical value calculated from an observation which is compared to a null distribution assess the significance of the observation, i.e. to ask whether it is likely that this value could be obtained by random sampling from the null model. For protein sequence alignment, the canonical example of a test statistic is the bit score of a BLAST alignment (Altschul *et al.* 1990), where the null model is alignment of the query to a random sequence. Reseek v2.0 uses alignment quality (*AQ*) as the test statistic:

(4) $$\begin{array}{c} t=\;\delta \Delta \;+\;\left(\alpha s-\beta s_{\mathrm{rev}}\right)/\left(L\;+\;\lambda \right),\;\\ AQ\left(Q,T|C\right)=1/\left[1+½10^{\left(\left[a+bt\right]/10\right)}\right]. \end{array}$$

Here, *Δ* is LDDT-mu, the variant of LDDT (Mariani *et al.* 2013) defined by Muscle-3D (Edgar *et al.* 2024); *s* is given by Equation (3); *L* = (|*Q*| + |*T*|)/2 is the mean length of the aligned proteins; *λ* is a parameter introduced to damp the growth in *t* for anomalously short queries; and *s_rev_* is the optimal alignment score of *Q* and *Q_rev_*, i.e. the Cα atoms of *Q* in reverse order. Parameters *α*, *β*, *γ*, *δ*, *λ, a* and *b* are constants trained on SCOP40 with hold-out validation. *AQ* was designed to give an intuitive interpretation analogous to the familiar *TM* score so that *AQ* ranges from zero to one and *AQ *<* *0.5 suggests a spurious alignment. Unlike *TM*, *AQ* is symmetrical between *Q* and *T*; it depends only on the alignment. An *E*-value is obtained from *AQ* by fitting to an empirical distribution (Supplementary Material).

The throughput of pair-wise comparisons is optionally accelerated by applying filters before proceeding to construct the alignment required to calculate *AQ* (details in Supplementary Material).

The optimal transformation for a rigid-body superposition implied by a local alignment is optionally constructed using the Kabsch algorithm (Kabsch 1976). The transformation is applied to atom coordinates for one of the structures to enable visualization of the superposition (Fig. 1).

**Figure 2.**
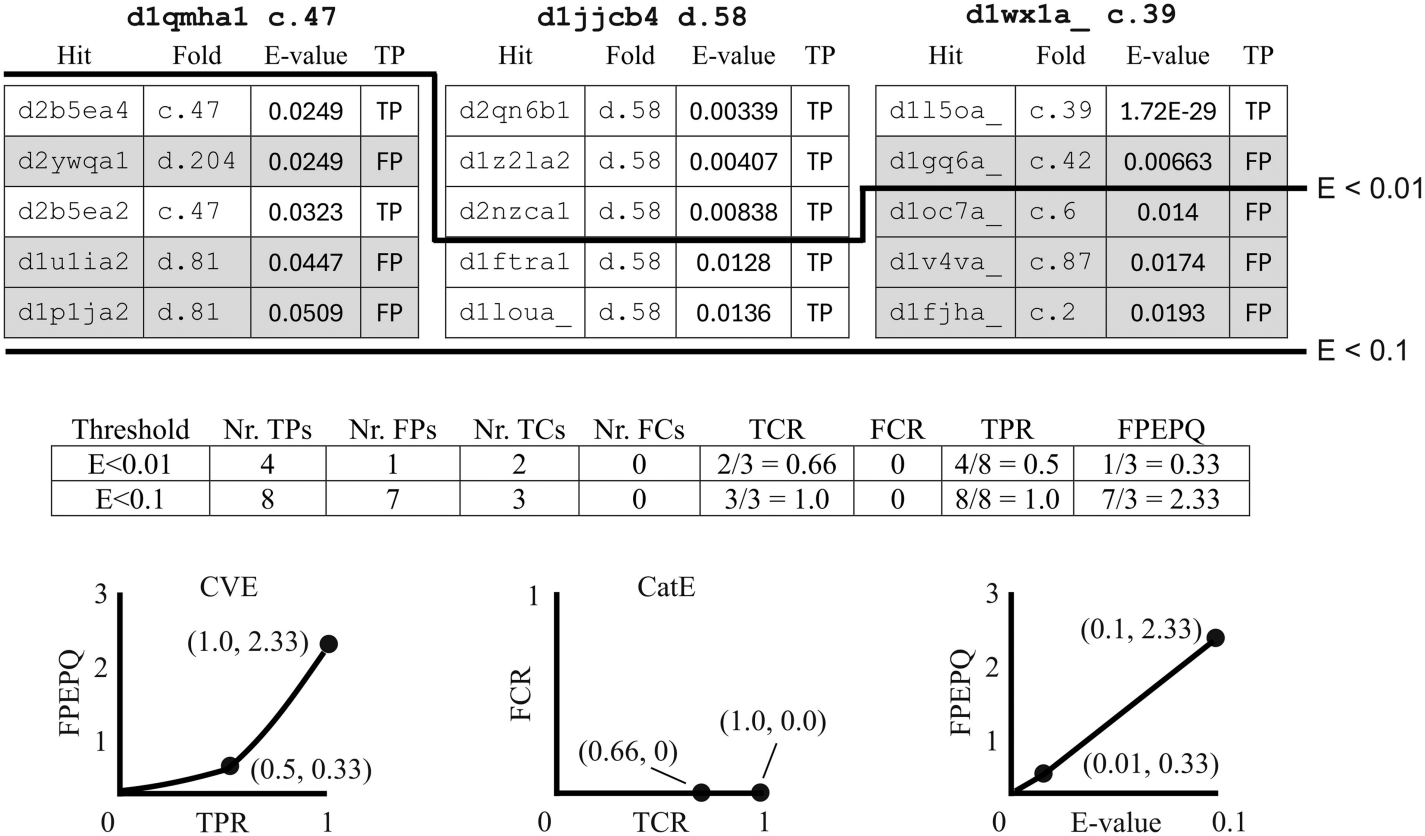
Measuring homolog detection and *E*-value accuracy on SCOP40. Here, accuracy metrics are calculated using the top five hits for three query structures d1qmha1, d1jjcb4, and d1wx1a with folds c.47, d.58, and c.39, respectively, sorted by increasing *E*-value. For this illustration, calculations assume that domains in the same fold are homologous, and that all homologs are included at *E *<* *0.1. At a given *E*-value threshold, the true positive rate (*TPR*, also called sensitivity) is the fraction of homologs found, and false-positive errors per query (*FPEPQ*) is the mean number of FPs. Plotting *FPEPQ* against *TPR* gives a Coverage versus Error (CVE) plot (lower-left) which shows the range of trade-offs that can be achieved by setting different *E*-value thresholds. Plotting *FPEPQ* against *E*-value (lower-right) measures the accuracy of *E* as a prediction of the number of errors; ideally *FPEPQ = E*. Here, *E*-values are under-estimated because there are 2.33 FP errors per query with *E *<* *0.1 and 0.33 at *E *<* *0.01. A CatE plot (lower-center) considers only the top hit. If it is above the threshold then it is a true category (TC) if correct or false category (FC) otherwise. See Supplementary Materials for further details and discussion.

**Figure 3.**
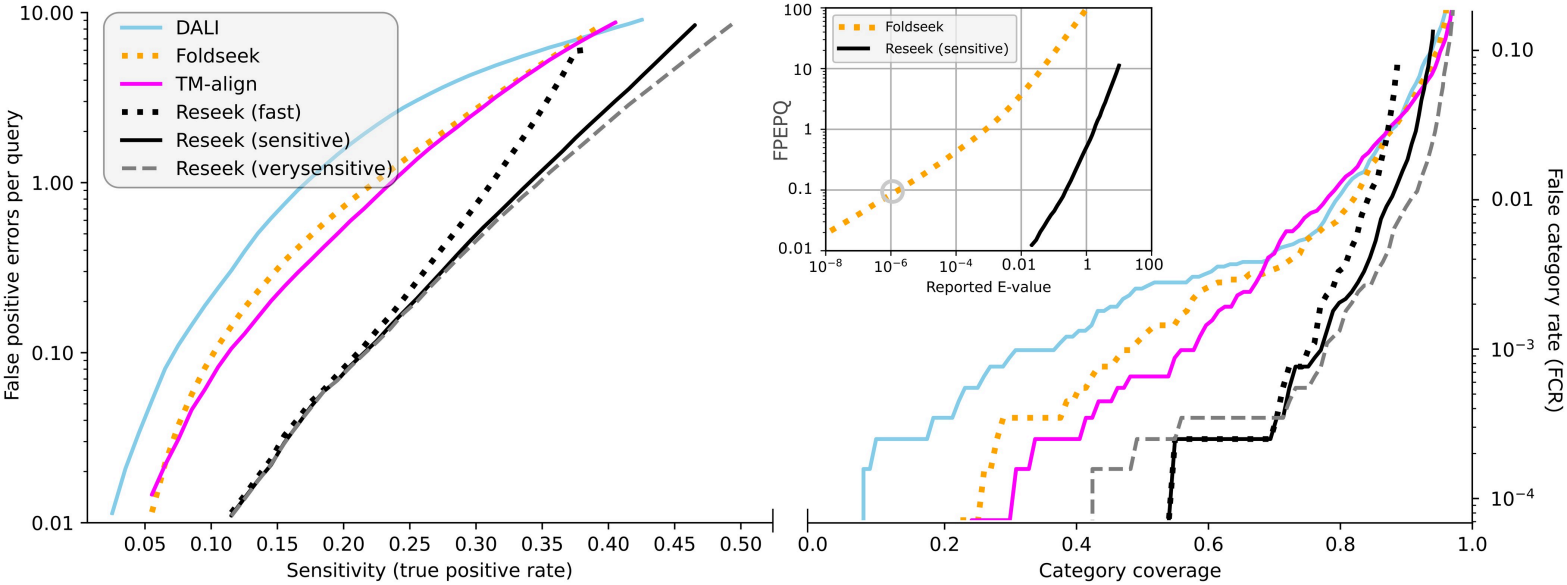
Sensitivity versus errors for homolog detection on SCOP40. The *x* axis is sensitivity as fraction of all homologs found above the threshold (left, CVE plot) or fraction of queries whose top hit above the threshold is homologous (right, CatE plot). The *y* axis is false-positive errors per query (*FPEPQ*) or false category rate (*FCR*) respectively, log scaling is used to highlight the very low error rates which are typically desired in practice. Higher accuracy is reflected by fewer errors at a given sensitivity, which gives a curve lower and to the right. Inset is reported *E-*value versus *FPEPQ*, which shows that Reseek *E*-values are in good agreement with measured error rates, while Foldseek *E*-values are underestimated. For example, at Foldseek *E*-value threshold 10^−6^ (circled), the measured *FPEPQ* is ∼0.1, i.e. five orders of magnitude higher than the estimate. See Supplementary Material for more plots.

Homolog detection performance was assessed on SCOP40. The SCOP database (Murzin *et al.* 1995) classifies protein domains superfamilies which are believed to be homologous. SCOP40 is a subset of SCOP such that the maximum a.a. sequence identity is 40%. SCOP40 v1.75 has 11 206 domains classified into 1960 superfamilies. Tested methods were used to make all-vs-all alignments of SCOP40 domains. Accuracy for a range of cutoffs is summarized by a coverage versus error plot (CVE) (Brenner *et al.* 1998) with sensitivity on the *x*-axis and *FPEPQ* on the *y*-axis (Figs 2 and 3). A CVE plot shows the sensitivity that can be achieved assuming that a cutoff is set by an ideal *E*-value (i.e. one that correctly predicts *FPEPQ*). Domains are considered to be homologous if they belong to the same superfamily, otherwise nonhomologous. I also report “CatE plots,” where sensitivity is the fraction of queries for which the top hit is a valid homolog. CatE plots model the common scenario where the goal is to assign the query to a functional category by assigning the annotation in the top hit(s). Here, I report CVE and CatE results for Reseek release v2.0, DALILite v5, TMalign 2022/4/12 and Foldseek 8-ef4e960. Other assessment methods and algorithms are reported in Supplementary Material.

The AlphaFold database (AFDB) (Varadi *et al.* 2022) has ∼200M predicted structures at the time of writing (20 000× larger), raising the issue of how to extrapolate SCOP40 results to large databases with AI-predicted folds. *FPEPQ* increases in proportion to database size (Altschul and Gish 1996), but the mean number TPs per query depends on the number of superfamilies in AFDB, which is not known. See Supplementary Material for discussion of extrapolating accuracy results to large databases.

## 3 Results

**Figure 4.**
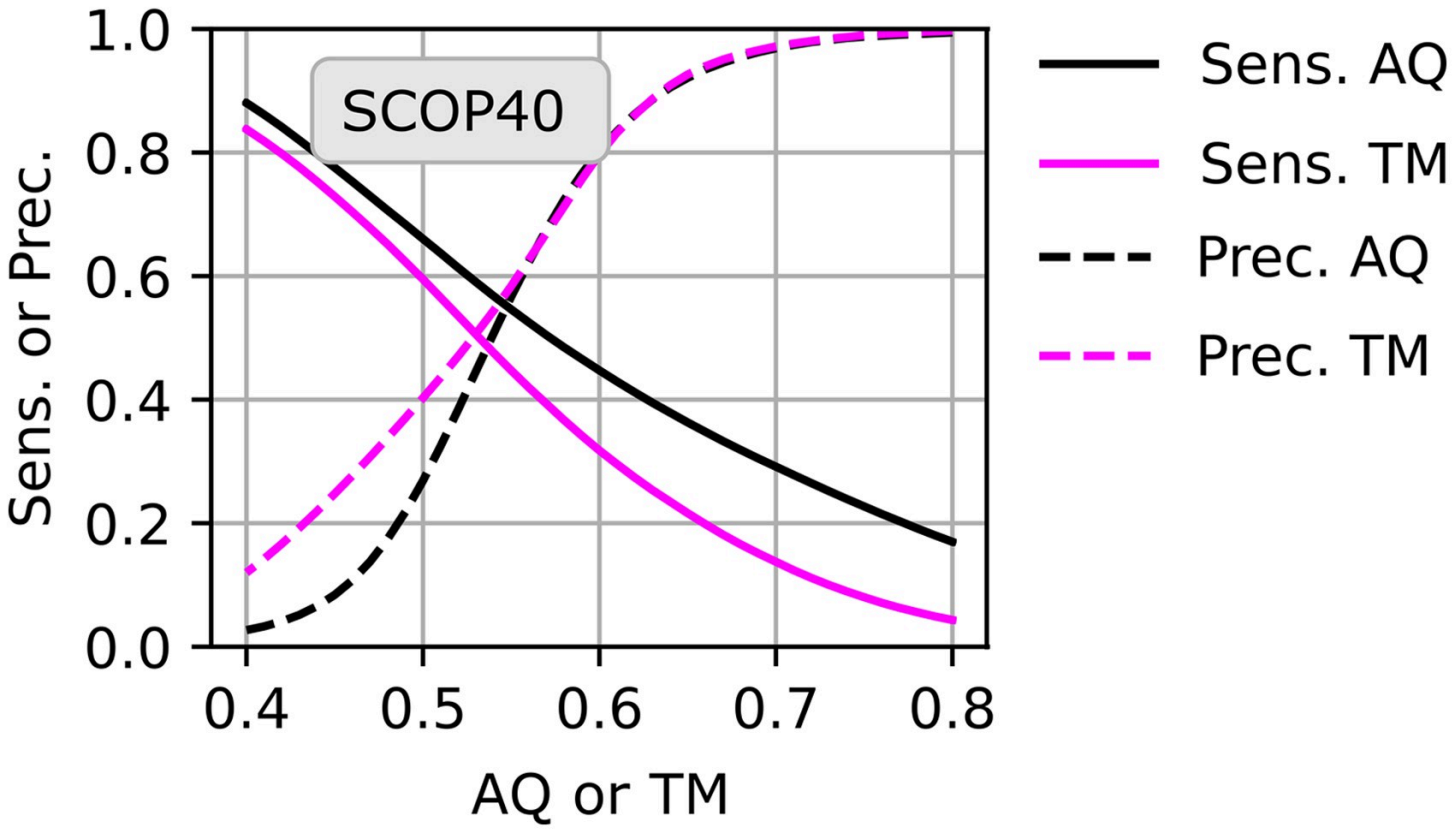
Precision and sensitivity as a function of *AQ* and *TM*. The *x* axis is the score, *AQ* for Reseek or *TM* for TMalign measured on SCOP40. The *y* axis is precision (dashed lines), i.e. the fraction of hits above this threshold that are TPs, or sensitivity (solid lines). The *TM *>* *0.5 threshold suggested for TMalign gives precision 0.4; using *TM *>* *0.6 or *AQ *>* *0.6 gives precision 0.8 which is likely to be more acceptable in practice. Note such guidelines require adjustment for large databases (Supplementary Material).

Performance on the SCOP40 test is shown in Table 1. The CVE plot in Fig. 3 shows *FPEPQ* from 0.01 to 10. The upper limit *FPEPQ *=* *10 corresponds to an ideal *E*-value threshold of 10 and therefore represents the high end of *E*-values typically used in practice, while *FPEPQ *=* *0.01 is the lowest error rate which can be reliably measured on SCOP40. These results show that Reseek has substantially higher homolog sensitivity versus the state of the art represented by DALI, TMalign, and Foldseek, with faster speed and lower memory use compared to Foldseek. Figure 3 (inset) shows *E*-value versus *FPEPQ* for Foldseek and Reseek; other tested methods do not report *E*-values. The Foldseek *E*-values are shown to substantially under-estimate error rates, while Reseek’s are in good agreement. Sensitivity and precision as a function of *AQ* and *TM* are shown in Fig. 4. For further results and discussion, see Supplementary Materials.

**Table 1.** Performance on the SCOP40 test.^a^

Method	Time	Memory	Sens(0.1)	Sens(1)	Sens(10)
BLASTP	127	35 Mb	0.090	0.11	0.13
DALI	4.1 × 10^5^	4.0 Gb	0.075	0.17	0.44
Foldseek	78	1.8 Gb	0.11	0.22	0.41
TMalign	3.7 × 10^5^	175 Mb	0.12	0.24	0.42
RS-fast	49	289 Mb	0.22	0.32	0.39
RS-sens.	178	339 Mb	0.22	0.34	0.48
RS-vs.	840	386 Mb	0.22	0.35	0.51

a Time is wall-clock elapsed search time in seconds on a 3.2 GHz Intel i9-14900K CPU (32 threads), excluding time to build the search database. Sens(0.1) is sensitivity at *FPEPQ *=* *0.1, similarly for Sens(1) and Sens(10). RS is Reseek with options -fast, -sensitive, and -verysensitive.

## 4 Discussion

The ubiquitous use of *E*-values implies that biologists prefer to control errors by setting a limit on the number of false positives. From this perspective, commonly used plots for assessing binary classifiers such as Receiver Operator Characteristic (ROC) (Fawcett 2006) and precision-recall (P–R) (Davis and Goadrich 2006) are problematic because they do not enable assessment of sensitivity at a given number of false positives and do not scale to large databases (see Supplementary Material for details and discussion). These issues were noted by the developers of SCOP40, who proposed the CVE plot as better suited to the assessment of homology search (Brenner *et al.* 1998), and this is now a well-established standard for benchmarking sequence search methods. However, to the best of my knowledge, CVE plots have not previously been used to assess protein structure search methods. Previously published benchmarks have used plots with accuracy metrics which conflate TPs and FPs (e.g. precision) and have adopted varying standards for determining true and false positives, some of which are poor models of practical search tasks; see Supplementary Material for review and discussion.

A well-estimated *E*-value is required to limit *FPEPQ*. As seen in Fig. 3, the Reseek *E*-value agrees very well with measured *FPEPQ*, while Foldseek radically over-estimates alignment significance, showing that Reseek is currently the only protein structure search algorithm reporting a robust estimate of expected errors.

In conclusion, Reseek achieves superior accuracy in homolog detection compared to the previous state of the art at search speeds comparable with Foldseek.

## Supplementary Material

btae687_Supplementary_Data

## Data Availability

The data underlying this article are available in the article and in its online supplementary material.
