# Supplementary material for: Protein structure alignment by Reseek improves sensitivity to remote homologs
Source: Bioinformatics. 2024 Nov 15;40(11):btae687. doi: 10.1093/bioinformatics/btae687 (PMC11601161; doi:10.1093/bioinformatics/btae687)
Supplement: btae687_Supplementary_Data [file btae687_supplementary_data.pdf]

Protein structure alignment by Reseek improves sensitivity to remote homologs

Robert C. Edgar

Supplementary Material

Contents

1 Structure features 2

2 MSP accelerator 3

3 K-mer accelerators 4

4 Goals for structure search benchmarks 4

5 Classifier accuracy 5

6 SCOP families, superfamilies and folds 6

7 Protein search as a binary classification task 6

8 Protein search as a categorization task 7

9 Category weighting 8

10 Sensitivity to the first false positive (SFFP) 9

11 Benchmarking structure search on SCOP 10

12 Extrapolating accuracy from SCOP40 to AFDB 14

13 Alignment correctness is not well-defined 15

14 Parameter training 15

15 Benchmarking and analysis code availability 16

References 16

1 Structure features

Here, i is the position of the C_α in the chain. If a feature is condensed to an alphabet with size L, it is denoted as $FeatureName(L)$. AA is the amino acid type, taking 20 possible values. $DistNEN$ is the distance in Angstroms between the C_α and its nearest Euclidean neighbor (NEN). "Euclidean" means 3D space, to emphasize the distinction from distance measured as number of residues along the chain. $DistREN$ is the distance in Angstroms between the C_α and its reverse Euclidean neighbor (REN), i.e. its nearest Euclidean neighbor considering only the opposite chain direction from its NEN). $Conf$ (local conformation) is a vector feature with all pair-wise distances between C_αs in the range $(i-\kappa)$ ... $(i+\kappa)$, i.e. the contact map for $2\kappa+1$ residues centered at i, with $\kappa = 3$ by default. This feature captures secondary structure; in particular, $Conf(3)$ correlates well with $\{$*helix, strand, other*$\}$ annotations by DSSP (Andersen and Rost, 2003), and is used to identify helix and strand elements for other features. $NENConf$ is the $Conf$ of the NEN. $RENConf$ is the $Conf$ of the REN. $NormDens$ is normalized density, a measure of how many residues are found in the Euclidean neighborhood surrounding the C_α, with more distant residues down-weighted. Density ($Dens$) is calculated as follows:

$$Dens = \sum_j e^{-d_{i,j}/D}, \quad (1)$$

where $D = 20$ Angstroms, $d_{i,j}$ is the Euclidean distance between position i and position j, and j is summed over positions from $(i-50)...(i+50)$ excluding positions $(i-3)...(i+3)$ as close neighbors in chain order are necessarily also close in Euclidean distance and therefore uninformative. Normalized density ($NormDens$) is calculated by re-scaling $Dens$ to a range zero to one, excluding outliers. $HelixDens$ is calculated by Eq. 1, including only positions which are alpha helices according to $Conf(3)$. $StrandDens$ is calculated by Eq. 1,, including only positions which are beta

strands according to $Conf(3)$. $DistNextHelix$ is the Euclidean distance between position i and the mid-point of the nearest alpha helix later in the chain than position i, excluding the helix which contains i if any. Secondary structure elements are identified as contiguous segments with the same $Conf(3)$. If there is no later helix, the distance is zero. $DistPrevHelix$ is similarly the Euclidean distance to the mid-point of the preceding alpha helix in the chain.

2 MSP accelerator

The throughput of pair-wise comparisons is optionally accelerated by applying filters before proceeding to construct the alignment required to calculate AQ. The MSP accelerator computes a similarity measure ω which should tend to be larger for more closely related structures:

$$\omega = MSP(Q,T;\mu) - MSP(Q_{rev},T;\mu), \tag{2}$$

where MSP is the maximum segment pair score, Q_{rev} is the chain of Q in reverse order, and μ is an alphabet chosen to balance the competing requirements of fast execution, which favors small alphabets, and effective filtering, which favors larger alphabets. MSP is the highest score which can be obtained by aligning a pair of contiguous subchains without gaps; it is calculated using a Smith-Waterman implementation with optimizations to exploit a scenario where the alphabet is small, both Q and Q_{rev} are likely to be compared with many different targets, gaps are not allowed, and explicit alignments are not required, only scores. In the prototype, μ is the 36-letter alphabet $\{Conf(3), NENConf(3), DistREN(4)\}$. A pair is discarded iff $\omega < \Omega$, where Ω can be chosen by the user. Smaller values increase speed, at the possible expense of reduced sensitivity.

3 K-mer accelerators

Other accelerators are based on k-mers, i.e. sub-chains of fixed length k C_αs, where a subset of $b \leq k$ letters may be considered according to a fixed pattern (Ma et al., 2002). K-mer indexes are widely used to accelerate searches in biological sequence applications (Marchet et al., 2021). The design of a k-mer index implies a complex trade-off between speed, sensitivity, and index size. To maximize speed, only exact k-mer matches (seeds) should be considered, because this enables the fastest lookups. The number of relevant exact seed matches may be optimized by varying k, b, the pattern of b letters within a seed, and the alphabet, and then seeking a design which maximizes sensitivity to related proteins while minimizing false positives. For U-sorting (Edgar, 2010), the correlation between E-value and number of unique seeds in common between pair of proteins should be maximized. Preliminary attempts were made to perform such optimizations for several types of seed index, all of which converged on the same seed design: $k = 11$, $b = 2$, $pattern = 10000000001$, with alphabet $\mu = \{Conf(3), NENConf(3), DistREN(4)\}$ (i.e, the same alphabet as the MSP accelerator). This design has $|\mu|^b = 1,296$ distinct seed values.

4 Goals for structure search benchmarks

Benchmark tests should strive to create realistic models of tasks performed by biologists in practice, and to report algorithm quality on these models using biologically relevant metrics. Search and alignment algorithms are used in many diverse ways, but I would suggest that the most typical use of searches is to classify a protein into a functional category. The user is hoping that the top hits belong to the same category (say, viral polymerases or CRISPR Cas-9s), in which case they can infer that the query belongs to this category. For such tasks, false positives (FPs) are a concern because valid hits are likely to be at most a tiny fraction of the search database, in which case a large

fraction of predicted hits may be FPs. Often there are no valid hits, in which case the top hit is certainly an FP, but this may be difficult to recognize if the first hit has high predicted significance. With sequence search by methods such as BLAST (Altschul et al., 1990) and hidden Markov models (Eddy, 2011), E-values (Altschul and Gish, 1996) have emerged as the gold standard for making a trade-off between sensitivity and errors. An E-value is an estimate of the expected number of FPs. If the user sets an E-value threshold of 10, then the expected number of FP hits for a given query is 10, and the expected total number of FPs for N queries is $10 \times N$. In my experience, $E = 10$ is the upper end of the range used in practice, users more typically set $E \ll 1$, say $E = 10^{-3}$ or $E = 10^{-6}$. To model this type of search task, a benchmark should enable assessment of sensitivity at error rates from 10 per query down to the smallest error rate which can be robustly measured. SCOP40 has 11,211 domains. An error rate of 0.001 per query corresponds to total of $0.001 \times 11,211 \approx 10$ errors summed over all domains. Ten errors out of $11,211^2 \approx 10^8$ alignments is too few to measure a frequency reliably, and $FPEPQ = 0.01$ (~100 errors) therefore represents a reasonable lower bound for error rates which can be robustly measured on this database.

The accuracy of E-values reported by an algorithm should be assessed because they are important in practice. If E-values are over-estimated, then sensitivity will be compromised unnecessarily, and conversely if E-values are under-estimated then the number of FPs may be substantially higher than the user desires.

5 Classifier accuracy

A bewildering variety of accuracy metrics have been proposed for classification tasks; see Table S1 for summary of notation and definitions used here. Unfortunately, terminology is inconsistent and different terms commonly found in the literature are equivalent; in particular, true positive rate (TPR), sensitivity, recall and coverage all

have the same definition, i.e. the fraction of all homologous pairs which are reported above a given threshold. The ubiquitous use of E-values implies that users care about the expected number of FPs regardless of the number of valid hits, suggesting that the number of errors should be considered separately rather than using a mixed metric that combines TPs and FPs. An example of a mixed metric is F_{max} which was used as the accuracy measure by (Holm, 2019). Other examples of mixed metrics include precision $= TP/(TP+FP)$, used in precision-recall plots, and false-positive rate $= FP/(TP+FP)$, used in ROC plots.

6 SCOP families, superfamilies and folds

SCOP classifies domains into a hierarchy with four ranks: class, fold, superfamily and family (Fig. S5). Domains assigned to the same superfamily are believed to be homologous. Domains in the same fold but different superfamilies are likely to be convergent but are sufficiently similar that homology cannot be ruled out. Families are somewhat arbitrary sub-categories of superfamilies into groups with similar function, where it is clear there is no dividing line between similar and dissimilar—any classification of protein function requires its own somewhat arbitrary hierarchy as in the Gene Ontology database (Gene_Ontology_Consortium, 2004). Folds are also somewhat arbitrary because structure similarity is subjective.

7 Protein search as a binary classification task

If the user sets a score or E-value threshold, then a protein search method can be considered as a binary classifier which predicts whether a given alignment is homologous (above the threshold) or non-homologous (below). Varying the threshold varies the trade-off between sensitivity and errors, which can be summarized in a plot with a measure of sensitivity on one axis and a measure that incorporates errors on the other. From this perspective, it is tempting to deploy popular plotting methods developed

for binary classifiers such as Receiver Operator Characteristic (ROC) (Fawcett, 2006) or precision-recall (Davis and Goadrich, 2006). However, these approaches conflate errors with TPs by using $FPR = FP/(TP+FP)$ and precision $= TP/(TP+FP)$, receptively, to characterize errors, and therefore they cannot be used to assess the regime with < 10 false-positive errors per query ($FPEPQ$) which is most relevant to typical biological search tasks. Also, unlike a typical binary classifier, hits below the cutoff are commonly considered to be undetermined rather than a prediction of non-homology. These issues were noted by a pioneering paper on the use of structure classifications to measure homology search accuracy (Brenner et al., 1998), which proposed the Coverage vs. Error (CVE) plot as better suited to assessment of homology search. A CVE plot shows coverage (fraction of homologs identified at a given threshold) as the x axis against $FPEPQ$ on a log-scaled y axis to clearly show sensitivity at very low error rates. Assuming an ideal E-value which is exactly equal to $FPEPQ$, a CVE curve enables the reader to find sensitivity at a given E-value by drawing a horizontal line from E on the y axis and noting the x value where this line intersects the curve. The actual numerical value of the cutoff corresponding to that point cannot be directly read from the plot; in my CVE analyses cutoffs can be found in an additional column in the tab-separated report files (https://github.com/rcedgar/reseek_bench).

8 Protein search as a categorization task

If a search is used to infer a structural or functional category, then it is the top reported hits that are most relevant because FPs further down the hit list belonging to other categories will typically be discarded by a biologist if the top few hits belong to the same category. Similarly, FNs may not be relevant providing the top hits are sufficient to assign the correct category. Modeling this approach in a benchmark test would require implementing a “top-hits” classifier capable of predicting a category from a list of hits. Such a classifier might differ from strategies used in practice and would itself require

validation, calling into question the credibility of the benchmark test as an independent standard. To avoid this problem, I propose a simplified model where the top hit alone is used to predict the category. By default, the category is SCOP superfamily as a proxy for homology. A score or E-value threshold is applied, as would be done in practice, so there may be no hits. To distinguish categorization from binary classification, TC and FC are used for true and false categories: a TC is a domain whose top hit is in the same category, and an FC is a domain whose top hit is in a different category. The true category rate (TCR) is the fraction of domains which are successfully classified of the domains which can be classified (i.e., excluding singleton domains), and the false category rate (FCR) is the fraction of domains for which the top hit is in a different category. This design ensures that an algorithm is not unfairly penalized for failing to enable top-hit classification when this is not possible, and conversely does penalize an algorithm for reporting an incorrect top hit regardless of whether a TP exists or not. The variation of TCR and FCR with cutoff is visualized as a Category Coverage vs. Error (CatE) plot where TCR is on the x axis and FCR is on a log-scaled y axis to clearly show the variation in sensitivity at lower error rates.

9 Category weighting

The sizes of SCOP40 superfamilies are highly variable, ranging from the largest superfamily `c.37.1` with 252 domains to 838 domains belonging to singleton superfamilies. It has been suggested (Green and Brenner, 2002) that superfamilies should be weighted by size so that they contribute equally, with two weighting schemes proposed. With linear weighting, a domain counts as $1/N$ where N is the superfamily size so that the total weighted number of domains in each superfamily is equal. With quadratic weighting, an alignment counts as $1/(N(N-1))$ so that the total weighted number of TP alignments for each superfamily is equal, excluding trivial self-alignments. While weighting seems reasonable, I do not use it here. I am already reporting a wider

range of different assessment metrics and plots that one would ideally want to see, and I don't want to add a further complication or test the reader's patience with more variants if this can be avoided. Also, I don't find the motivation for weighting to be compelling; it is not clear to me why category weighting could give a meaningful improvement in the ability of the benchmark test to predict accuracy in practice. Databases used in practice, including PDB (Berman et al., 2002) and AFDB (Varadi et al., 2022), contain unbalanced categories. Also, the weighting proposal was made in in 2002 when SCOP was comparable in size to the full PDB database and thus could be considered a good model of typical searches made in practice. Today, practical searches are often performed on millions of structures predicted by AI with the consequence that extrapolating from performance on SCOP40 to performance in practice is much more challenging. The small improvement that might be achieved by weighting is surely insignificant compared to the uncertainty in extrapolation.

10 Sensitivity to the first false positive (SFFP)

SFFP (Van Kempen et al., 2024) is calculated by sorting alignments for each SCOP40 domain separately and reporting the number of TP hits found above the first FP as a fraction of the total TPs for this domain in the database. SFFP is called ROC1 in (Van Kempen et al., 2024); I use an alternative notation here to avoid confusion with ROC curves. SFFP can be interpreted as an attempt to model a "top hit" strategy via a different simplification from CatE. However, I believe that SFFP is unrealistic in three important respects. First, a user will almost always set a cutoff, but SFFP does not. For example, if the E-value of a TP is 100 then a biologist would probably not believe it, but SFFP nevertheless counts it as a successful detection. Second, it neglects that in practice queries may have no TP hits, which is modeled in SCOP40 by singletons, i.e., domains which are the only member of their family, superfamily or fold. The top hits for a singleton are necessarily FPs and are therefore not counted by SFFP regard-

less of their scores or E-values. For example, if the top hits to a singleton are FPs with E~10^{-9} then a biologist is likely to believe they are valid, but SFFP will ignore them and thus fail to penalize the algorithm. Third, if the domain is not a singleton, but the algorithm reports a FP as the top hit, this case should be considered an error but is penalized only as a loss in sensitivity.

The results reported here show that SFFP correlates poorly with other measures of accuracy which I believe are more informative for practical applications. For example, DALI has substantially higher SFFP compared to Reseek while Reseek is consistently superior to DALI by other metrics; e.g. on superfamily DALI has SFFP=0.48 which is ~$1.5\times$ Reseek-sensitive (0.31) (see Table S3), but at $FPEPQ = 1$ coverage of Reseek-sensitive is 0.34 which is ~$2\times$ better than DALI (0.17). From an algorithm developer's perspective, SFFP is informative because it separates the problem of obtaining a good sort order from the problem of calculating a test statistic or E-value which is commensurate between queries. However, from a user's perspective, a commensurate measure of significance is essential and SFFP is therefore flawed for benchmarking.

11 Benchmarking structure search on SCOP

Table S2 summarizes the truth standards and plots reported in published protein structure search benchmark tests using SCOP. Here, the six identified standards are referred to by short names **Fam1** ... **Fold6** given in Table S2. In my opinion, most of these truth standards are poor models of searches performed by biologists in practice.

Fam1 considers hits to the same family as TPs, yet discards all hits to different families except for very distant hits to different folds which are considered FPs. If family is a meaningful category, and a biologist might search for this category, then a hit to a different family should be considered a false positive. The most important false

positives to a biologist would typically be closely related families because these are the most likely to be mixed with true positives near the top of a sorted list of hits, but these are ignored by the standard—there is a vast gulf between a different family and a different fold, but this gulf is entirely ignored by **Fam1**. To give a concrete example, suppose a biologist is searching for SWIRM domains (family `a.4.1.18`) and a structure search algorithm reports SLIDE domains (`a.4.1.13`, same superfamily), homeodomains (`a.4.1.1`, same superfamily) and ribosomal protein S18 domains (`a.4.8.1`, same fold) mixed together with SWIRM domains above the chosen threshold, then the algorithm will be rewarded for the correct hits but not penalized for any of the hits to these different families. Also, family is an arbitrary, subjective category (see "SCOP families, superfamilies and folds"), analogous to using taxonomic genus. It would not be appropriate to consider genus as a category for generic structure search benchmarking because the optimal score cutoff will depend on which genus you are searching for, and in the same way it is not appropriate to use SCOP family.

SF4 considers hits to different families in the same superfamily to be TPs, yet ignores hits to the same family. The use of family to define categories for benchmarking is again inappropriate (see above). If superfamily is a meaningful category, and a biologist might search for it, then hits to the same family should also be considered true positives. Also, hits to a different superfamily in the same fold are ignored, creating a gulf between true positives and false positives analogous to the gulf in **Fam1**.

Fold5 considers hits to different superfamilies in the same fold to be true positives yet ignores hits to the same superfamily. As with **Fam1** and **SF4**, this standard uses essentially different categories to define true positives vs. false positives. It could be

viewed as asking the question “does the search algorithm sort convergent folds above dissimilar folds if there are no homologs in the search database?”. This is a reasonable question to ask, but it is misleading to report results as TPs/FPs for a classifier because it is not possible to build a practical classifier based on this standard. If the biologist is interested in searching for similar folds, regardless of whether they are homologous, then homologs should be included as TPs (**Fold6**). On the other hand, if the biologist is specifically interested in convergent folds, then homologs must be removed first—this is the scenario modeled by **Fold5**, but the problem of identifying homologs is not solved in practice with sufficient accuracy. Therefore, **Fold5** is not a realistic model of a practical search task.

Fold6 assesses fold as the category, using natural definitions that same fold is TP and different fold is FP. Fold as a category is somewhat subjective, but this is unavoidable in a structure classification hierarchy. This standard is clear and consistent, and in my view is more reasonable than **Fam1**, **SF4** and **Fold5**. The main point to note about **Fold6** is that true positives may be convergent domains with unrelated function. Regardless of convergence vs. homology, the biological relevance of hits to different superfamilies is questionable for most practical applications because function at fold rank is usually so different as to be of little value. For example, all-alpha fold `a.60` is “SAM domain-like”, which comprises 16 superfamilies including `a.60.9` “lambda integrase-like, N-terminal domain”, `a.60.10` “Enzyme I of the PEP:sugar phosphotransferase system HPr-binding (sub)domain”, and `a.60.13` “Putative methyltransferase TM0872, insert domain”. As seen in these examples, SAM-like domains exhibit a wide range of functions involving interactions with proteins, DNA or RNA, and knowing only that a domain belongs to fold `a.60` therefore gives limited insight into its functional role.

SF3 is the original standard set by the authors of SCOP (Brenner et al., 1998; Green and Brenner, 2002). Hits to the same superfamily are TPs, hits to different folds are FPs, and hits to different superfamilies in the same fold are ignored. This is the most conservative standard in the sense that measured TPs are most likely to be true according to an objective (albeit not fully known) criterion, and measured FPs are most likely to be false according to the same criterion. However, choosing this standard may nevertheless give a misleading assessment of homology detection accuracy because of the large number (581,772) of alignments which are ignored compared to the number of TPs (454,766). An ideal algorithm which is able to accurately distinguish homologous from convergent folds will be heavily penalized by this standard because the most challenging cases are discarded.

SF2 assesses superfamily as the category, using natural definitions that same superfamily is TP and different superfamily is FP. This is my preferred standard here; it has not previously been used in the literature to the best of my knowledge. **SF2** implicitly assumes that SCOP successfully assigns homologs to the same superfamily. If this assumption is correct, **SF2** is self-evidently the best standard for homology detection. If in fact a minority of superfamilies are homologous and should be merged, then **SF2** remains a better approximation to homology detection than **SF3** or any of the other proposed standards above. The only scenario where another standard would be preferred is when a majority of superfamilies should be merged, in which case **Fold6** would better reflect homology. In my view, it is most likely that a minority of superfamilies should be merged. Even if it were true that most superfamilies should be merged, **SF2** might still better reflect the goals of practical searches, because the functions of proteins in different superfamilies are rarely similar enough to be biologically informative

(see discussion of **Fold6** above), and from this perspective superfamily would be a meaningful and useful category despite splitting of homologous groups into multiple superfamilies. From these considerations, I believe that **SF2** is the best single standard for assessing homology detection accuracy.

12 Extrapolating accuracy from SCOP40 to AFDB

See Figs. S3 and S4. Subscript S indicates SCOP40 and A indicates AFDB. Let Δ be the increase in number of structures (~$20,000$), σ be the increase in number of superfamilies (unknown, probably $1 < \sigma < 10$ with $\sigma = 4$ as a rough guess for illustration), and N_{SF} be the number of structures in superfamily SF. NT, the total number of true positives excluding trivial self-hits, is $NT = \sum_{SF} N_{SF}(N_{SF} - 1)$. Thus, NT depends on the number of superfamilies (SFs) and on their size distribution. Assume a similar size distribution for simplicity, then $NT_A = (\Delta/\sigma)NT_S$, and at a given score cutoff, $FP_A \approx \Delta FP_S$, $TP_A \approx (\Delta/\sigma)TP_S$, $Sensitivity_A \approx Sensitivity_S$, and $FPEPQ_A \approx \Delta FPEPQ_S$.

If the goal is to set a cutoff based on estimated $FPEPQ$, then a score cutoff tuned on SCOP40 must be adjusted to reduce the expected number of false positives by a factor of Δ, i.e. ~$20,000$.

Precision cannot be extrapolated directly because TP_S and FP_S scale by different factors, so the shape of a Precision-Recall plot will change in a non-intuitive way. Similarly for a ROC plot because the false-positive rate does not scale. By contrast, with a CVE plot both axes scale, though the scaling factor for the x axis (sensitivity) is not known. However, the relative performance of different search algorithms can be extrapolated with confidence—if algorithm X is substantially better than algorithm Y on a CVE plot for SCOP40, it will also be more accurate on AFDB.

13 Alignment correctness is not well-defined

As described in (Edgar and Tolstoy, 2024), I believe that structural alignments are inherently ambiguous and an objective standard for alignment correctness is therefore not possible in principle. While metrics such as TM score and Z score can be useful and informative, I do not believe they are appropriate for comparative validation because any given metric is biased towards algorithms which attempts to maximize the same metric or similar—using TM will favor TM-align, using Z will favor DALI, and so on. Therefore, I do not attempt comparative validation of alignment accuracy *per se* in this work.

14 Parameter training

The Reseek algorithm has a large number of parameters, including G_{open}, G_{ext}, β, γ, λ, the features to include ($\mathbf{F}$) as a subset of all implemented features ($\mathbf{F}^*$), and weights of the included features $\{w_f,\ f \in \mathbf{F}\}$. In version 2, 11 features were implemented, giving $2^{|\mathbf{F}^*|} = 2^{11} = 2,048$ possible choices for $\mathbf{F}$. For each possible $\mathbf{F}$, there are $|\mathbf{F}|$ weights and four scalar parameters G_{open}, G_{ext}, β and λ. The following protocol was implemented to enable practical tuning of this large parameter space. A given subset of features and their proposed weights were first evaluated using training alignments by constructing a log-odds score matrix. Higher expected score of the matrix (ES, also known as relative entropy (Altschul, 1991)) was used as the objective. For a given subset and its weights, ES can be calculated from a large number of preconstructed alignments in a fraction of a second, enabling exploration of many parameter combinations. First, all pairs of features from $\mathbf{F}^*$ were evaluated with a range of relative weights, keeping the best few feature pairs and their best weights, i.e. those with highest ES. For each such pair and its weights, all possibilities for adding a third feature were explored, again with a range of weights for the new feature. This procedure was iterated

until a point of diminishing returns was reached by adding new features. This gave a pool of promising combinations for further consideration. The next stage was to evaluate candidate parameter sets by constructing ungapped alignments on a SCOP40 subset with high $SFFP$ as the objective. Small variations in the weight of each parameter were tried one at a time, keeping changes that improved $SFFP$, repeating with increasingly small variations until convergence was reached. Finally, gapped alignments were generated to optimize G_{open}, G_{ext}, α, β, γ and λ using a similar procedure to weights, i.e. by introducing variations one parameter at a time with high $SFFP$ as the objective. The size of the default mega-alphabet is $h = 20 \times 16^8 = 85,899,345,920$ letters.

Reseek's large parameter space raises potential concerns of over-fitting. To investigate this, I performed two-fold cross-validation by splitting families in SCOP40 into two equal-size subsets A and B. Parameters were trained separately on A and B, then used to measure $SFFP$ and $Sens(1)$ on both subsets. As seen in Table S4, results from self-training and cross-training are almost identical, showing that over-fitting is minimal. This justifies the use of default parameters trained on all SCOP40, which on the basis of these cross-validation results I would expect to generalize slightly better than parameters trained on a subset.

15 Benchmarking and analysis code availability

Scripts and raw analysis results are deposited at https://github.com/rcedgar/reseek_bench.

References

Altschul, S. F. (1991). Amino acid substitution matrices from an information theoretic perspective. *Journal of molecular biology*, 219(3):555–565.

Altschul, S. F. and Gish, W. (1996). [27] local alignment statistics. In *Methods in enzymology*, volume 266, pages 460–480. Elsevier.

Altschul, S. F., Gish, W., Miller, W., Myers, E. W., and Lipman, D. J. (1990). Basic local alignment search tool. *Journal of molecular biology*, 215(3):403–410.

Andersen, C. A. and Rost, B. (2003). Secondary structure assignment. *Structural bioinformatics*, 44:339–363.

Berman, H. M., Battistuz, T., Bhat, T. N., Bluhm, W. F., Bourne, P. E., Burkhardt, K., Feng, Z., Gilliland, G. L., Iype, L., Jain, S., et al. (2002). The protein data bank. *Acta Crystallographica Section D: Biological Crystallography*, 58(6):899–907.

Brenner, S. E., Chothia, C., and Hubbard, T. J. (1998). Assessing sequence comparison methods with reliable structurally identified distant evolutionary relationships. *Proceedings of the National Academy of Sciences*, 95(11):6073–6078.

Davis, J. and Goadrich, M. (2006). The relationship between precision-recall and roc curves. In *Proceedings of the 23rd international conference on Machine learning*, pages 233–240.

Eddy, S. R. (2011). Accelerated profile hmm searches. *PLoS computational biology*, 7(10):e1002195.

Edgar, R. C. (2010). Search and clustering orders of magnitude faster than blast. *Bioinformatics*, 26(19):2460–2461.

Edgar, R. C. and Tolstoy, I. (2024). Muscle-3d: scalable multiple protein structure alignment. *bioRxiv*, pages 2024–10.

Fawcett, T. (2006). An introduction to roc analysis. *Pattern recognition letters*, 27(8):861–874.

Gene_Ontology_Consortium (2004). The gene ontology (go) database and informatics resource. *Nucleic acids research*, 32(suppl_1):D258–D261.

Green, R. E. and Brenner, S. E. (2002). Bootstrapping and normalization for enhanced evaluations of pairwise sequence comparison. *Proceedings of the IEEE*, 90(12):1834–1847.

Holm, L. (2019). Benchmarking fold detection by dalilite v. 5. *Bioinformatics*, 35(24):5326–5327.

Ma, B., Tromp, J., and Li, M. (2002). Patternhunter: faster and more sensitive homology search. *Bioinformatics*, 18(3):440–445.

Marchet, C., Boucher, C., Puglisi, S. J., Medvedev, P., Salson, M., and Chikhi, R. (2021). Data structures based on k-mers for querying large collections of sequencing data sets. *Genome research*, 31(1):1–12.

Van Kempen, M., Kim, S. S., Tumescheit, C., Mirdita, M., Lee, J., Gilchrist, C. L., Söding, J., and Steinegger, M. (2024). Fast and accurate protein structure search with foldseek. *Nature Biotechnology*, 42(2):243–246.

Varadi, M., Anyango, S., Deshpande, M., Nair, S., Natassia, C., Yordanova, G., Yuan, D., Stroe, O., Wood, G., Laydon, A., et al. (2022). Alphafold protein structure database: massively expanding the structural coverage of protein-sequence space with high-accuracy models. *Nucleic acids research*, 50(D1):D439–D444.

Supplementary Tables and Figures

Term / symbol	Meaning
t	Threshold, minimum score or maximum *E*-value
Category	Family, superfamily or fold.
TP	Nr. true positives above *t*
TC	Nr. true categories above *t*, i.e. number of domains for which the top hit is in the same category.
FP	Nr. false positives above *t*
FC	Nr. false categories above *t,* number of domains for which the top hit is in a different category.
FN	Nr. false negatives above *t*
ND	Nr. domains in SCOP40
NS	Nr. singleton domains in SCOP40, i.e. domains which are the only member of its category.
NT	Nr. homologous pairs in SCOP40, excluding self-pairs<br>= maximum possible TP
NF	Nr. non-homologous pairs in SCOP40
NP	Total nr. pairs in SCOP40, excluding self-pairs<br>= ND (ND - 1)<br>= NT + NF
TPR<br>= Sensitivity<br>= Recall<br>= Coverage	True positive rate<br>Fraction of all homologous pairs reported above *t*<br>= TP / NT<br>= TP / (TP + FN)
Precision	Fraction of hits above *t* which are correct<br>= TP / (TP + FP)
FPR	False positive rate<br>Fraction of hits above *t* which are errors<br>= FP / (TP+ FP)
FPEPQ	Mean false positive errors per query above *t*<br>= FP / ND
TCR	True Classification Rate, fraction of non-singleton domains whose top hit is in the same category<br>= TC / (ND - NS)
FCR	False Classification Rate, fraction of domains whose top hit is in a different category<br>= FC / ND
SFFP	Sensitivity to the first false positive<br>= fraction of all possible TPs found above the first FP, considering the list of hits for each domain separately.

Table S1. Definitions and notation.

TP	FP	Ignored	NT	NI	Standard
Same family	Different Fold	Different family in same SF, or different SF in same fold	108,718	927,820	**Fam1**
Same SF	Different SF	(None)	454,766	0	**SF2**
	Different fold	Different SF in same fold	454,766	581,772	**SF3**
	Different fold	Same family, or different SF in same fold	346,048	690,490	**SF4**
Same fold	Different fold	Same SF	1,036,538	454,766	**Fold5**
	Different fold	(None)	1,036,538	0	**Fold6**

Reference	Fig. / Tbl.	Standard(s)	Wt.	Plot
van Kempen et al. 2024	Fig. 2<br>Fig. S5	**SF4**<br>**Fam1 SF4 Fold5**	Yes	P-R [1]
Margelevičius 2024	Fig. 5	**Fam1 SF3 Fold6**	No	P-R
Holm 2019	Tbl. 1<br>Tbl. S1	**Fold5**<br>**Fam1 SF4 Fold5**	No	(None)
Green & Brenner 2002	Fig. 7 (a)<br>Fig. 7 (c)	**SF3**<br>**SF3**	No<br>Yes	CVE
Brenner et al. 1998	Fig. 5	**SF3**	No	CVE

[1] Some plots in (van Kempen et al. 2024) appear to be calculated incorrectly (see https://github.com/steineggerlab/foldseek-analysis/issues/5)

Table S2. Truth standards for protein structure search in the literature. All benchmarks used SCOP40 except (Holm, 2019), which used 140 domains as a query set against SCOP clustered at 70% a.a. identity. TP: criteria for true positives, FP: criteria for false positives, Ignored: criteria for discarding hits, NT: total TPs in SCOP40 all-vs-all, NI: total ignored, Standard: short name for the standard used here, Plot: type of plot (P-R=precision-recall, CVE=coverage-vs-error), Wt.: category weighting used Yes or No.

Method	SFFP
BLASTP	0.10
Reseek-fast	0.23
Reseek-sensitive	0.31
Foldseek	0.39
Reseek-verysensitive	0.42
TM-align	0.45
DALI	0.48

Table S3. Sensitivity to first false positive (SFFP). Values are measured on SCOP40 using **SF2** as truth standard.

Test	Train	Sens(1)
A	A	0.600
A	B	0.600
B	B	0.538
B	A	0.537

Table S4. Two-fold cross-validation of Reseek training on SCOP40. Families in SCOP40 were divided into two equal-sized subsets A and B. Algorithm parameters were optimized separately for A and B. This table reports $Sens(1)$, i.e. TPR at $FPEPQ = 1$, for all four possible combinations of training set and test set, showing that self-training gives almost identical results to cross-training. This shows that the training procedure does not over-train parameters to SCOP40.

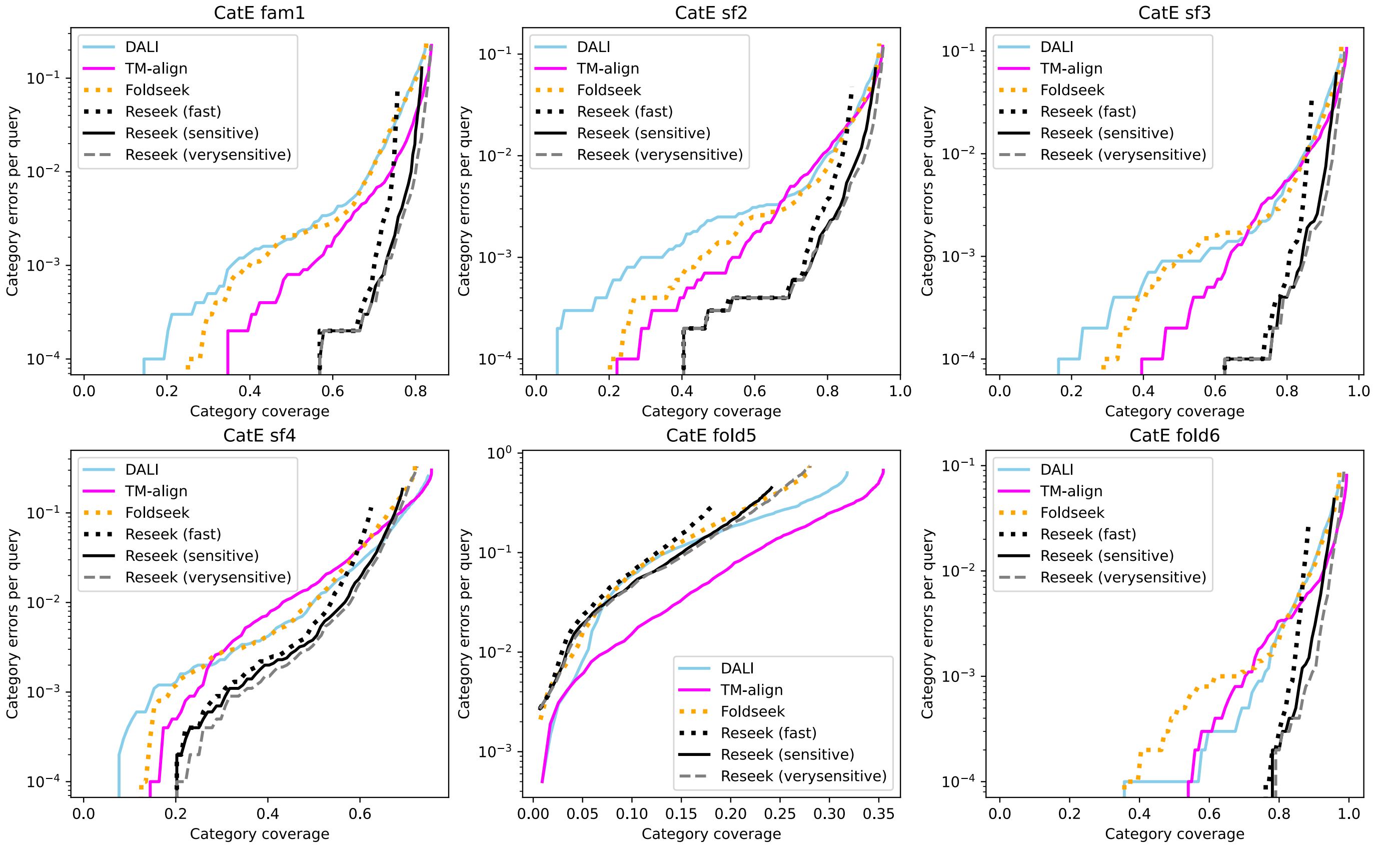

Figure S1. **Category vs. Error (CatE) plots.** CatE plots are shown using the six truth standards from Table S2 The middle-top plot uses **SF2** (superfamily category with no hits ignored) which reproduces the right-hand pane of Fig. 3. On these tests, Reseek exhibits clearly superior accuracy over most of the coverage range except for **Fold5**, which excludes hits to the same suprerfamily while using fold to determine FPs. The biological relevance of **Fold5** is unclear.

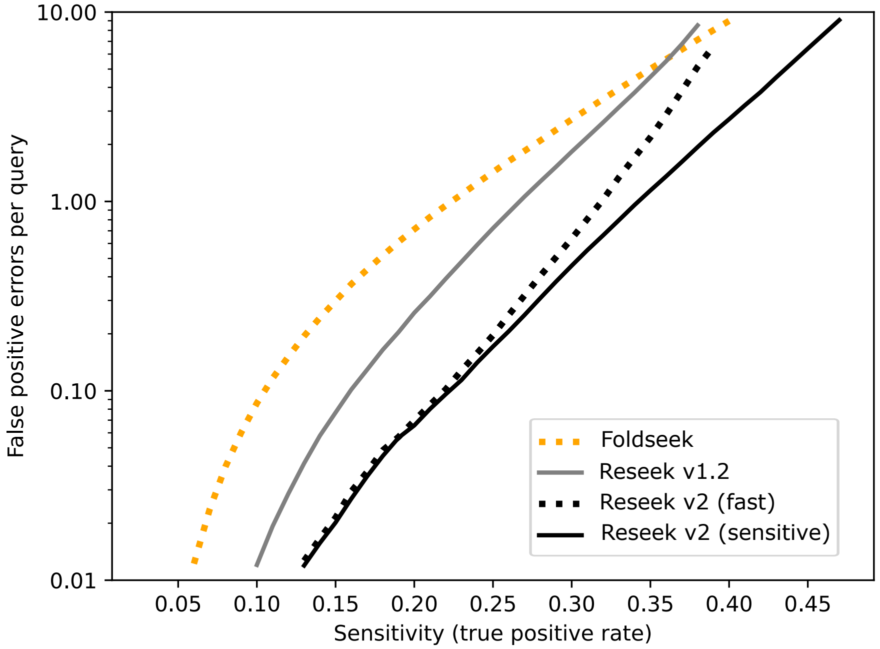

Figure S2. **Improvement in Reseek v2 over v1.2 on superfamily CVE**. Foldseek is also shown for comparison. Reseek v1.2 was described in preprint https://www.biorxiv.org/content/10.1101/2024.05.24.595840v2.

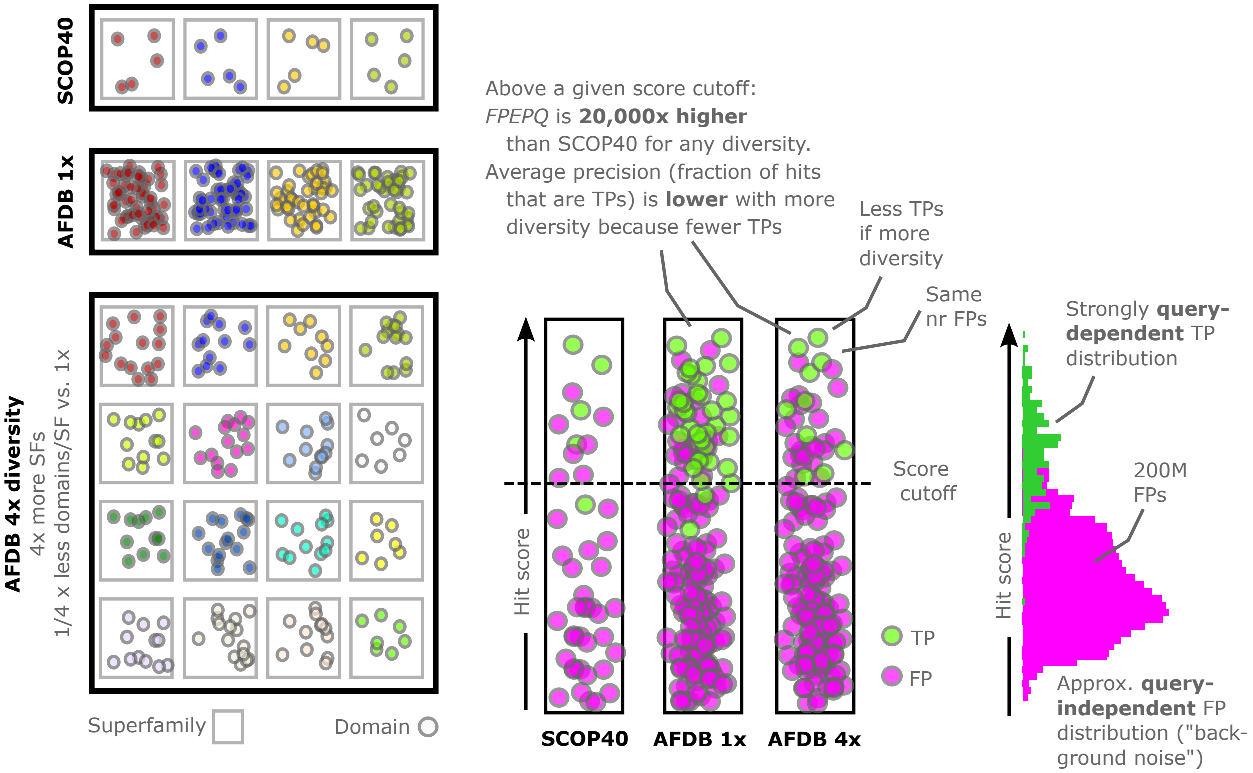

Figure S3. Extrapolating accuracy from SCOP40 to AFDB. AFDB is $20,000\times$ larger than SCOP40. At a given cutoff, $FPEPQ$ increases in proportion to the database size. The rate of false positives as a function of alignment score or E-value is approximately independent of the query and can be predicted as "background noise" (Fig. S4). By contrast, true positives strongly depend on the query, and the average number of true positives depends on the diversity of the database, i.e. how many superfamilies it contains and their abundance distribution. If AFDB has the same number of superfamilies as SCOP40 and has similar abundance distribution ($1\times$ diversity), then the number of TPs and FPs at a given cutoff are both $20,000\times$ larger and extrapolation is straightforward. However, AFDB contains many previously unsolved protein families, and therefore likely contains substantially more superfamilies. Here, I use $4\times$ as an educated guess for illustration. To set a threshold at an estimated $FPEPQ$, the score cutoff must be adjusted to reduce the number of FPs by a factor of $20,000\times$ compared to SCOP40. This can be accomplished by setting the same E-value threshold on both databases, providing that the E-value is estimated accurately.

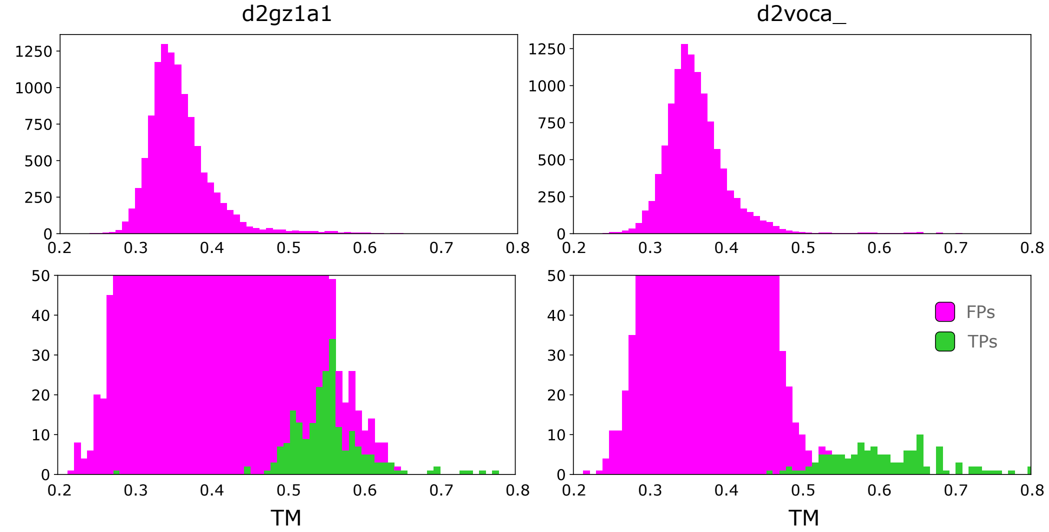

Figure S4. TP and FP distributions for two example domains. Distribution of SCOP40 TP and FP scores for domains d2gz1a1 and d2voca_, using superfamily as the standard for both TPs and FPs. Here, TM score is used; similar results would be seen for the Reseek AQ score, DALI Z score etc. Scores are placed in 100 bins from TM=0 to TM=1 to plot the histogram. The upper histograms show the full range where the TPs are too small to be visible. In the lower histograms the y axis is truncated to show the TPs. Notice that the background distribution of FPs has similar shape and similar mode for both domains, this is typical and can be exploited to estimate E-values. However, the distribution of TPs is quite different, with the mode shifted leftwards in d2gz1a1 so that the bulk of the distribution overlaps the FP distribution. This illustrates that the TP distribution is query-dependent, and may be entirely absent if the query has no homologs in the database. To scale these distributions to AFDB, the FP histogram bars would increase in size by ~$20,000\times$ while the TP bars depend on the query, as in SCOP40, and also on the diversity of AFDB relative to SCOP40. For example, if AFDB has $4\times$ more superfamilies, then on average the TP bars would increase by a factor of $5,000\times$ rather than $20,000\times$ (Fig. S3).

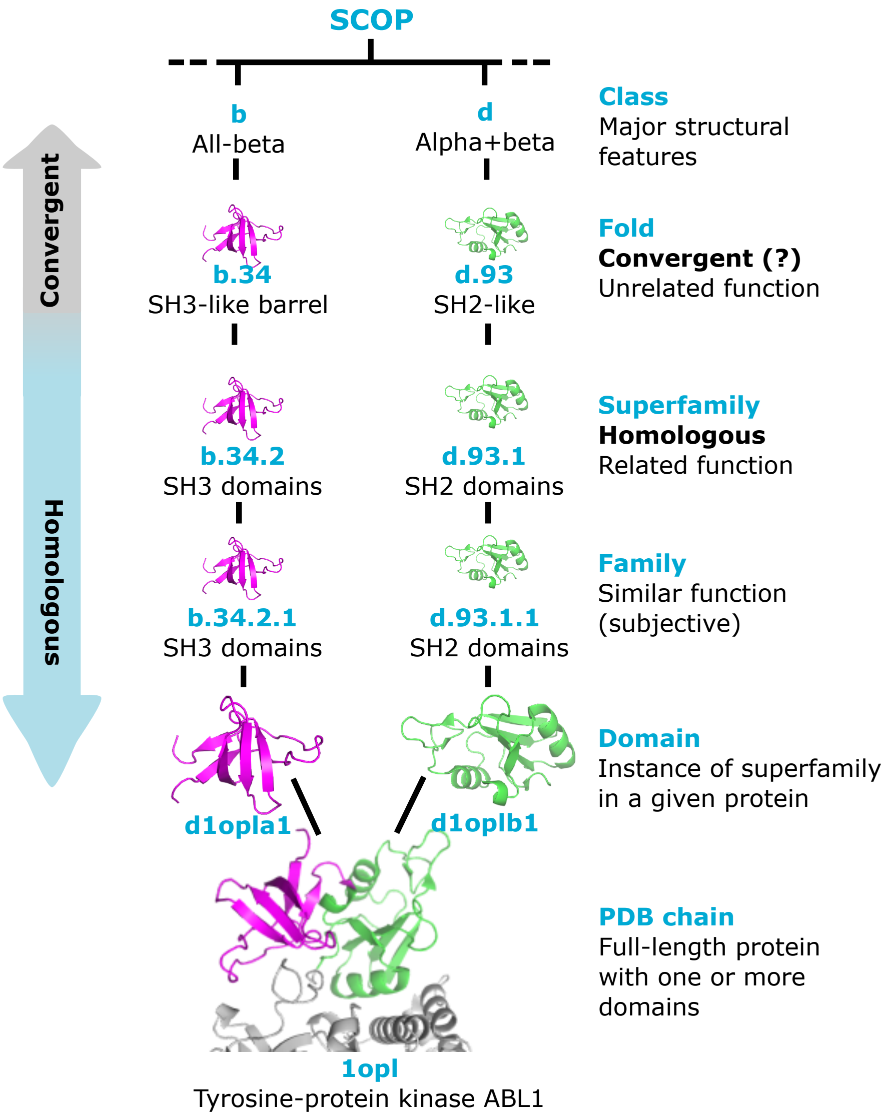

Figure S5. SCOP hierarchy. SCOP classifies protein domains into folds, superfamilies and families. Superfamilies are believed to be homologous. Different superfamilies in the same fold lack evidence for homology and are therefore likely to be similar due to convergence, though homology cannot be ruled out. Families indicate similar function. Family and fold categories are somewhat arbitrary, because similarity of fold and similarity of function are both subjective judgments where reasonable people might disagree, especially for family where it is clear there is no dividing line between similar and dissimilar – any classification of function requires its own somewhat arbitrary hierarchy as in the Gene Ontology database. Functions of different superfamilies in the same fold are typically so diverged as to be effectively unrelated from a genome annotation perspective.

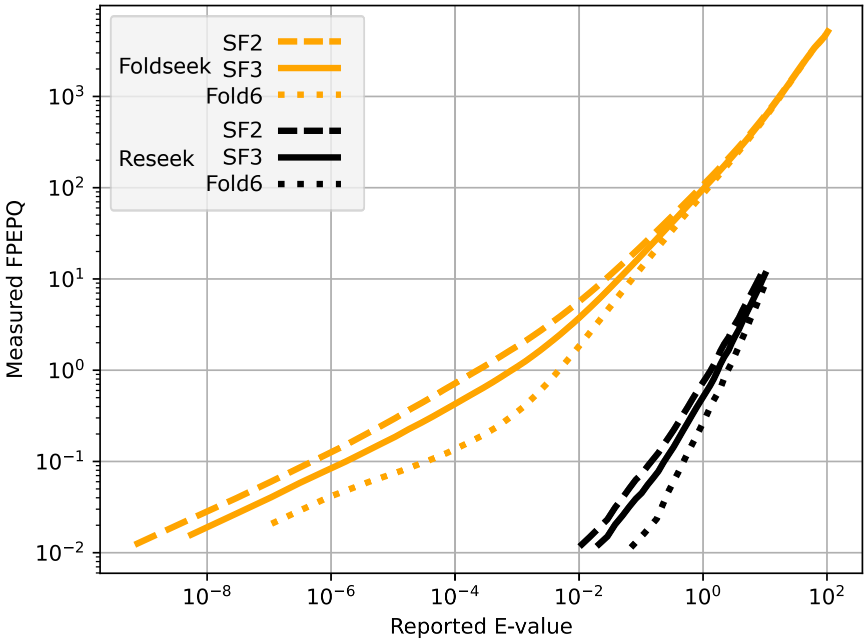

Figure S6. ***E*-value accuracy of Foldseek and Reseek.** *E*-value (estimated *FPEPQ*) is plotted against measured *FPEPQ* using **SF2**, **SF3** and **Fold6** truth standards. By all three truth standards, Reseek *E*-values are in good agreement with measured *FPEPQ* while Foldseek *E*-values are severely under-estimated.

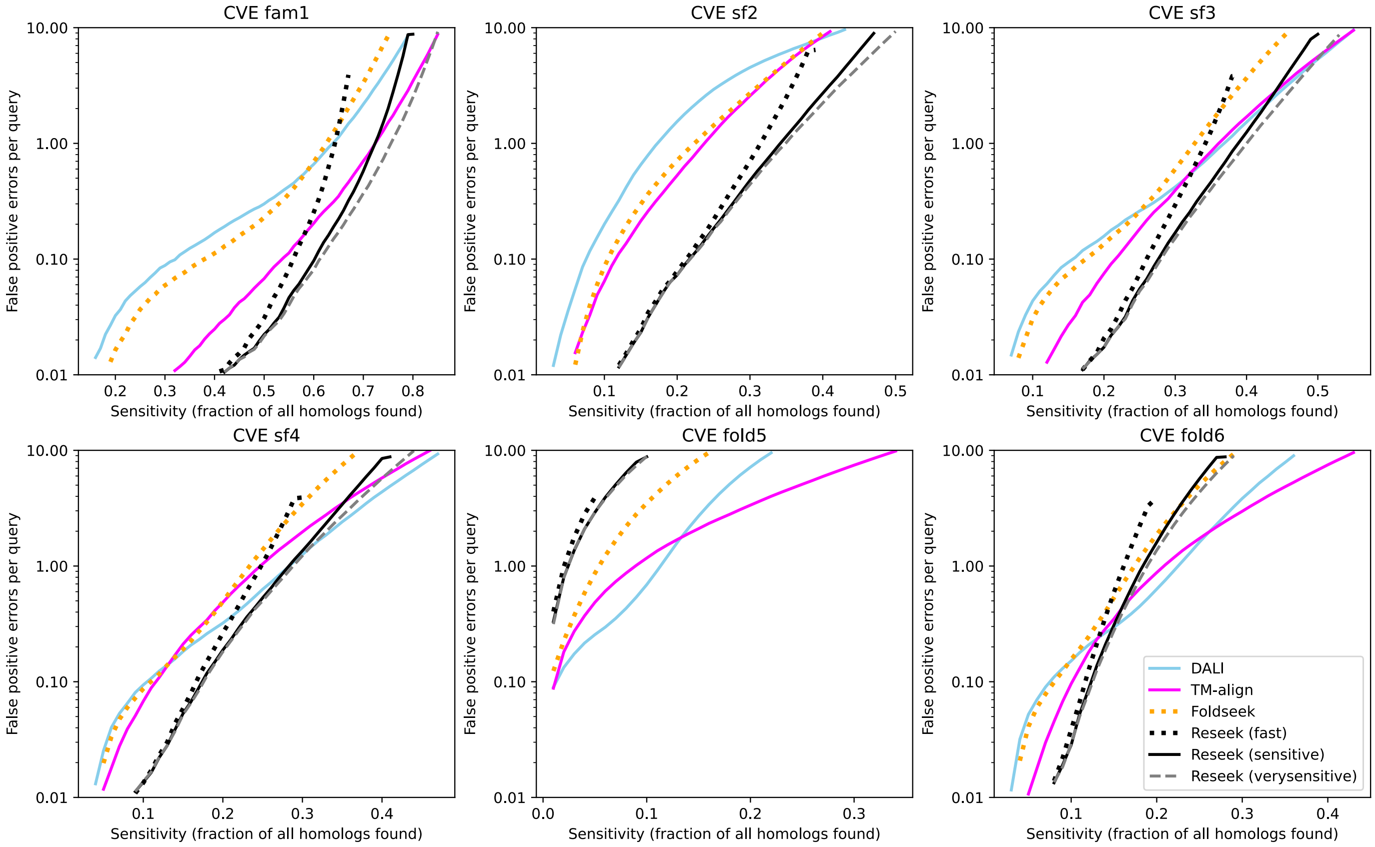

Figure S7. **Coverage versus error plots.** CVE plots are shown for the six truth standards of Table S2. The preferred standard, **SF2**, is reproduced in Fig. 3 of the paper. At lower $FPEPQ$ values corresponding to E-values typically used in practice, Reseek has higher sensitivity by all standards except **Fold5** (hits to the same superfamily ignored).

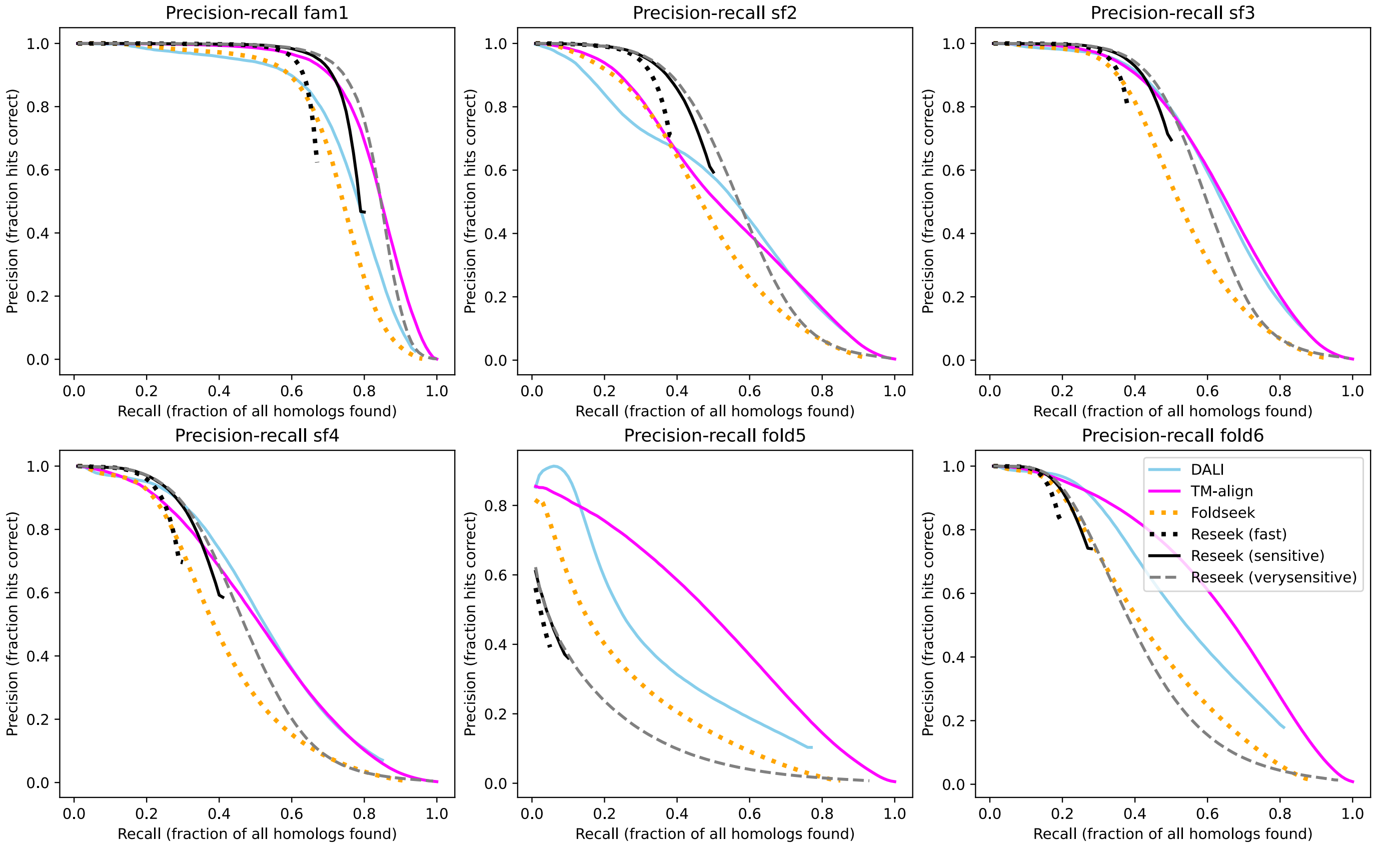

Figure S8. Precision-recall (P-R) plots P-R plots are shown for the six truth standards of Table S2. P-R and ROC plots are deprecated for benchmarking homology detection because they do not enable assessments at effective E-value cutoffs and do not scale to larger databases.

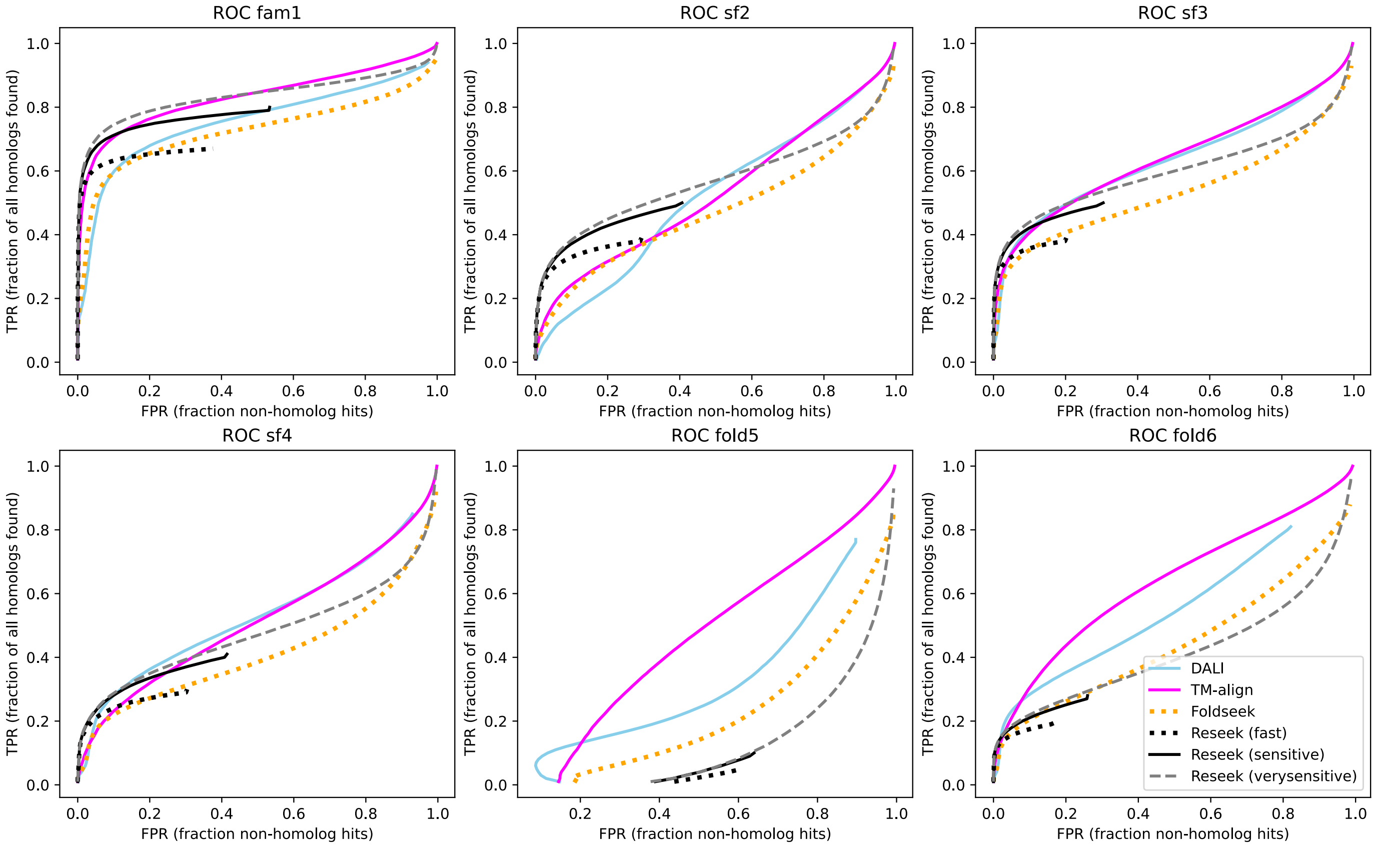

Figure S9. Receiver-operator characteristic (ROC) plots ROC plots are shown for the six truth standards of Table S2. P-R and ROC plots are deprecated for benchmarking homology detection because they do not enable assessments at effective *E*-value cutoffs and do not scale to larger databases.

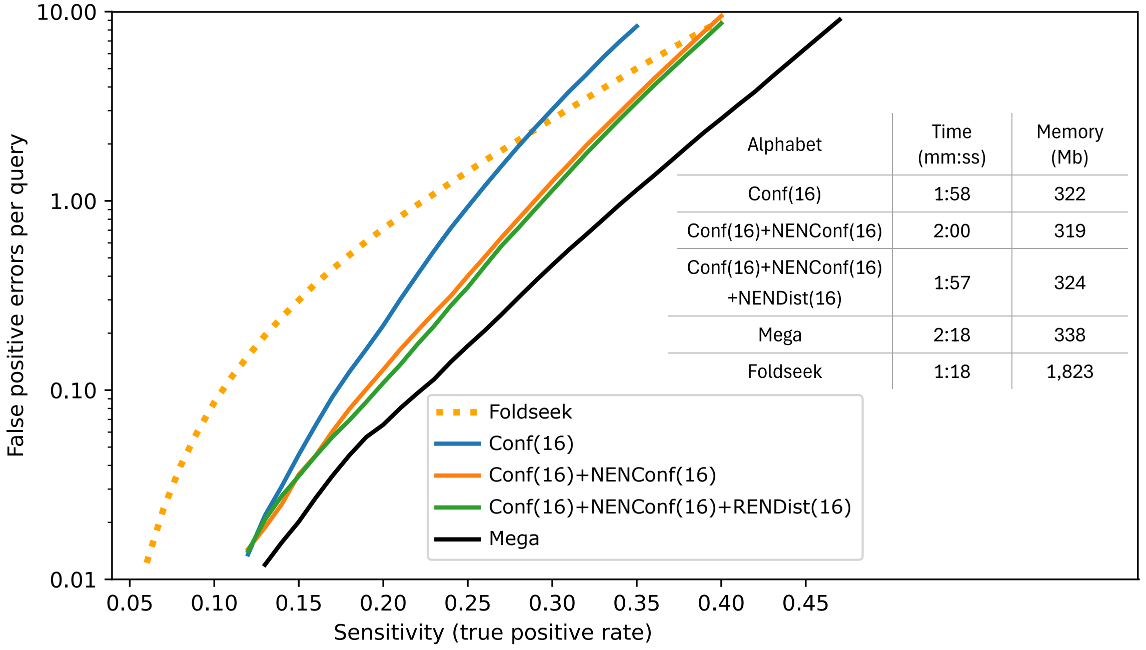

Alphabet	Time (mm:ss)	Memory (Mb)
Conf(16)	1:58	322
Conf(16)+NENConf(16)	2:00	319
Conf(16)+NENConf(16) +NENDist(16)	1:57	324
Mega	2:18	338
Foldseek	1:18	1,823

Figure S10. **Performance of Reseek with alphabet size.** Performance on Mega is compared with three smaller alphabets on a SCOP40 CVE plot (**SF2** truth standard) with elapsed time and memory shown in the inset table. All alphabets include a 20-letter amino acid feature. Uniform feature weights were used; higher accuracy can probably be obtained by optimizing weights. The -sensitive option was used for Reseek. Foldseek performance is also shown for comparison.

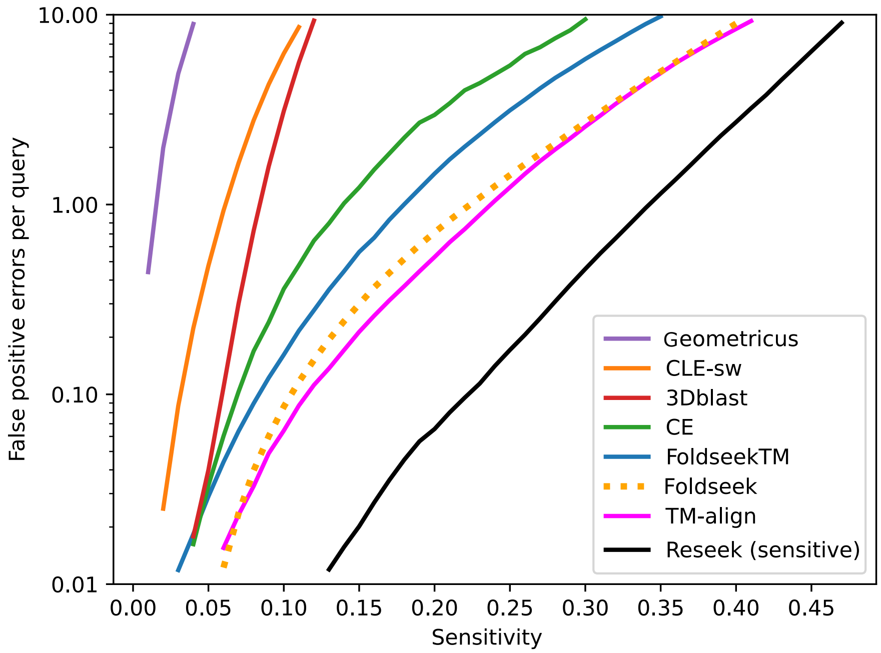

Figure S11. Assessment of other methods by superfamily CVE. Performance of less accurate methods is compared to Foldseek, TM-align and Reseek-sensitive using the **SF2** standard from Table S2. FoldseekTM is TM score reported by Foldseek, causing Foldseek to optimize superpositions which increases runtime by orders of magnitude.
